# Diets within Environmental Limits: The Climate Impact of Current and Recommended Australian Diets

**DOI:** 10.3390/nu13041122

**Published:** 2021-03-29

**Authors:** Bradley Ridoutt, Danielle Baird, Gilly A. Hendrie

**Affiliations:** 1 Commonwealth Scientific and Industrial Research Organisation (CSIRO) Agriculture and Food, Clayton, Victoria 3169, Australia; 2 Department of Agricultural Economics, University of the Free State, Bloemfontein 9300, South Africa; 3 CSIRO Health and Biosecurity, Adelaide 5000, Australia; danielle.baird@csiro.au (D.B.); gilly.hendrie@csiro.au (G.A.H.)

**Keywords:** climate change, dietary guidelines, diet quality, discretionary food, greenhouse gas emissions, GWP*, life cycle assessment, planetary boundaries, sustainable diet

## Abstract

Planetary boundaries are an important sustainability concept, defining absolute limits for resource use and emissions that need to be respected to avoid major and potentially irreversible earth system change. To remain within the safe operating space for humanity, there is a need for urgent adoption of climate-neutral diets, which make no additional contribution to warming. In the first study of its kind, a new climate metric, the Global Warming Potential Star (GWP*), was used to assess greenhouse gas (GHG) emissions associated with 9341 Australian adult diets obtained from the Australian Health Survey. Dietary climate footprints averaged 3.4 kg CO_2_-equivelent per person per day, with total energy intake explaining around one quarter of the variation. Energy-dense and nutrient-poor discretionary foods contributed around one third. With lower climate footprint food choices, a diet consistent with current Australian dietary guidelines had a 42% lower climate footprint. Currently, it is not possible to define a climate-neutral dietary strategy in Australia because there are very few climate-neutral foods in the Australian food system. To bring Australian diets into line with the climate stabilization goals of the Paris Agreement, the most important need is for innovation across the agricultural and food processing industries to expand the range of climate-neutral foods available.

## 1. Introduction

The production of food for human consumption has been estimated to contribute between 19% and 29% of total global greenhouse gas (GHG) emissions [1]. As such, the food system has become an important focal point for GHG emissions reduction [2,3,4]. While the food system is responsible for a wide range of environmental impacts, including impacts from water and land use and from the application of agricultural chemicals such as fertilizers and pesticides, GHG emissions reduction has received the most attention [5,6], probably due to the global implications for planetary health [7] and the interactions between climate change and many other environmental concerns [8]. On the one hand, action is needed to reduce GHG emissions associated with food production, processing, packaging and distribution. However, there is also scope for the adoption of dietary patterns that are less GHG emissions-intensive [9] as part of a movement toward more sustainable lifestyles as described in Sustainable Development Goal 12 [10].

There are now numerous studies reporting the GHG emissions of different self-reported and conceptual dietary patterns, many of which conclude that reducing the intake of livestock products, especially ruminant livestock products (beef, lamb and goat meat, as well as dairy foods), is an important strategy to reduce diet-related GHG emissions [5,11,12]. However, of concern is that many dietary patterns with lower GHG emissions have been found to be linked to poor nutritional and health indicators [13,14,15]. As such, concerns about the contribution of energy-dense/nutrient-poor discretionary or indulgence foods to total dietary GHG emissions have also been raised [5]. These foods make little contribution to meeting nutritional needs, yet they add to total dietary GHG emissions. In addition, they can contribute to overconsumption of dietary energy which is another way they can inflate dietary GHG emissions. Total dietary energy intake and total dietary GHG emissions have been found to be positively correlated [5]. It is therefore critically important that interventions to achieve the lowering of dietary GHG emissions are developed alongside well-established public health nutrition objectives relating to health, well-being and reducing the incidence of diet-related disease [16]. There is also a need to situate dietary GHG emissions reduction goals within the context of global targets for climate action. The Paris Agreement is the primary global consensus for action to combat the threat of climate change [17]. This instrument, now ratified by 187 states, sets the ambitious goal of limiting global mean temperature rise to below 1.5 °C above preindustrial levels. The issue is that with most dietary GHG emission studies it is unclear whether results are consistent with these climate stabilization goals. Studies may indicate that one dietary pattern has lower GHG emissions than another. However, rarely are diets benchmarked against global GHG emissions reduction targets.

The assessment of dietary GHG emissions is complicated by the variety of different GHGs and their diverse climate impacts. The main GHG emissions associated with food production are carbon dioxide (CO_2_), nitrous oxide (N_2_O) and methane (CH_4_). These GHGs differ in both atmospheric lifetime and radiative efficiency, which describes the greenhouse strength of a gas [18]. To assess the combined climate impact of a basket of GHGs, a climate metric is typically applied, which establishes equivalence between the different gases. In the great majority of dietary GHG emission studies the 100-year Global Warming Potential (GWP100) climate metric is used [5], reporting the cumulative contribution to radiative forcing over a future 100-year time horizon. However, the GWP100 climate metric is one of many different climate metrics and the use of alternative climate metrics, using different criteria for equivalence between the different GHGs, can lead to greatly different interpretations of the relative importance of different foods. While GWP100 has become a kind of default climate metric, the Intergovernmental Panel on Climate Change, the world’s preeminent body assessing the evidence of climate change, has expressly stated that the GWP100 climate metric should not be considered to have any special significance [18]. Importantly, dietary GHG emissions reported using the GWP100 climate metrics cannot be simply used to determine alignment with temperature-based climate stabilization targets, as expressed in the Paris Agreement [19].

Recently a new climate metric has been introduced, known as GWP* [20,21]. This metric is designed to compare the temperature response from a change in rate of emission of a short-lived GHG with the temperature response from a pulse emission of CO_2_. As such, the metric is especially relevant to the evaluation of diets because methane, which is a comparatively short-lived GHG with an atmospheric lifetime of around 12 years [18], is one of the important emissions from the food system. In contrast, CO_2_ emissions are highly persistent, with an impact on the climate system lasting potentially for millennia [22,23,24,25]. The very long-term climate impact of CO_2_ is the reason why climate stabilization depends on actions to achieve net zero emissions of CO_2_. With short-lived GHGs, climate stabilization is possible with a steady emissions profile over time. Agricultural studies have begun to explore GWP* in terms of the contribution of livestock production to global warming, but we are not aware of any application yet in regard to dietary intake.

In this study, we evaluate the climate impact of 9341 self-reported Australian daily diets using the GWP* climate metric. To enable the identification of healthier diets with lower climate impact we also estimated the healthiness of diets using a diet quality score. Our objective was to quantify the gap, if any, between current Australian diets and the goal of climate-neutral diets which make no additional contribution to global temperature increase. In addition, we assess whether a dietary pattern consistent with the current Australian dietary guidelines [26] aligns with climate-neutral food consumption. To our knowledge, this is the first study to assess a large number of individual diets using the GWP* climate metric.

## 2. Materials and Methods

### 2.1. Dietary Intake Data

Dietary intake data were obtained from the National Nutrition and Physical Activity Survey component [27] of the Australian Health Survey [28] as described previously [29,30,31]. In brief, the dietary intake data were collected for 9341 adults by means of a 24-h recall process administered by the Australian Bureau of Statistics (ABS) using trained interviewers. A complex sampling method was used, enabling the estimation of dietary intake for the Australian adult population, as well as demographic subgroups, through the application of population weighting factors. To enable comprehensive analysis of nutrient intakes, the data describe more than 5000 individual foods and beverages consumed on the day prior to the interview, as well as the portion sizes. Data were collected over a 13-month period to account for seasonal variations in eating habits and across all days of the week. As with all dietary intake surveys, there is the possibility of inaccurate recall or intentional misrepresentation of foods and portion sizes eaten. To facilitate use of the dietary intake data, the ABS has published estimates of the prevalence of under-reporting [28] that were used to uniformly adjust the data. This adjustment was necessary to avoid systematic underestimation of the climate impacts of food consumption and to enable reliable comparison of reported and recommended diets.

In order to integrate the dietary intake data with the climate impact data, processed foods and mixed dishes were disaggregated into their basic components and cooked food portions were translated into raw quantities. This task was undertaken during previous studies [29,30,31] and the same approach was used. For each individual daily diet, total energy intake was determined using data obtained from the Australian Food Composition Database [32], along with the number of serves of each of the food groups described in the Australian Dietary Guidelines [26]. Beverages that are not included within the official food groups, such as tea and coffee, formed a separate group. Different fresh meats and alternatives were separately studied as meats have been reported to be important sources of dietary GHG emissions [33,34,35,36,37]. Different discretionary choices were also separately examined because they are widely overconsumed by Australians and are a major public health nutrition concern [38,39,40]. Discretionary choices are energy-dense and nutrient-poor foods and beverages high in saturated fat, added sugars and salt, and alcohol [26]. By applying the ABS population weighting factors, the mean dietary intake for Australian adults was calculated. In addition, mean values were calculated for the age and gender subgroups described in the Australian Dietary Guidelines, namely 19 to 50 years, 51 to 70 years and 71 years and above [26].

### 2.2. Diet Quality Assessment

For each individual daily diet, a diet quality score was calculated by applying the Dietary Guideline Index [41] as described previously [29,30,31]. This index, ranging from 0 to 100, describes the degree of compliance of an individual diet with the food-based Australian Dietary Guidelines [26]. A higher score reflects greater compliance.

### 2.3. Climate Impact Assessment

The climate impacts of individual foods were assessed using the GWP* climate metric [20,21], which is an adaptation of the GWP100 climate metric designed to express equivalent future warming. With this approach, emissions of long-lived GHGs, such as CO_2_ and N_2_O, are treated in the same way. However, emissions of short-lived GHGs, such as methane, are treated differently, whereby the contribution to future warming is determined by the change in rate of emission over time by the responsible agricultural sector. Details of the calculation process are documented in the associated references [20,21,42]. For this study, GWP* results for Australian livestock products (beef meat, sheep meat, chicken meat, pig meat, eggs and milk) were obtained from a previous study [43] where the specific calculation parameters are described. GWP* results for Australian rice were calculated following an identical approach (see Table S1).

The climate impacts of foods without significant methane emissions associated with their production were compiled as follows. For cereals, oilseeds, legumes, sugar, banana and almond, GHG emissions data for Australian production were obtained from the Australian Life Cycle Inventory Database [44]. For other fresh foods, where nationally representative life cycle inventory data are not available in Australia, GHG emissions data were obtained from a recent systematic review [45]. For processed commodities not generally grown in Australia, such as tea leaves, coffee beans and cocoa beans, GHG emissions data were obtained from the Ecoinvent LCA Database [46]. As described previously [30,31], conversion factors were used to translate agricultural commodities into retail products and edible portions. GHG emissions associated with food processing were derived from a survey of energy use in generic food processing operations (Supplementary Table S2) using GHG emission factors for electricity and fuel combustion sourced from the Australian Government [47]. The global warming potentials used in the study were 1, 265 and 28 for CO_2_, N_2_O and CH_4_, respectively [18]. Results obtained using the GWP* climate metric are reported as a climate footprint in the unit kg CO_2_-e (equivalent). A complete list of climate footprint results for foods used in this study is presented in Supplementary Table S3. This study did not consider GHG emissions associated with food packing as this information was not collected during the dietary intake survey and the same foods can be packaged in a variety of ways. Similarly, the study did not include GHG emissions associated with kitchen storage and preparation of meals for the same reasons.

### 2.4. Dietary Pattern Modelling

Three dietary patterns were modelled for the largest demographic subgroup described in the Australian Dietary Guidelines [26], that is, the 19 to 50 year old age grouping (*n* = 5157). Firstly, quadrant analysis was used to identify a sub-group of self-reported diets with higher diet quality and lower climate impact (i.e., better diets; *n* = 1068). In performing the quadrant analysis, daily diets within 0.25 standard deviations of the mean of each parameter were excluded. Secondly, a recommended diet was constructed by scaling the average 19 to 50 year old diet so that the recommended number of servings of each food group were achieved. The Australian Dietary Guidelines [26] emphasize the benefits of choosing a wide variety of foods from the five food groups. However, they are not prescriptive about specific food choices. For example, they do not specify which vegetables should be eaten or which types of lean meat or protein alternatives. As such, it is possible to construct a range of possible diets that are equally compliant with the recommendations. This second dietary pattern was based on the same types of foods as the average 19–50 year old Australian reported to consume, but with portion size adjusted to meet the recommended number of serving of each food group. Thirdly, a recommended diet was constructed based on the “better diets” described above, which included lower climate impact food choices within a food group.

### 2.5. Correlation Analysis

The Pearson correlation coefficient was used to evaluate the relationship between dietary energy intake and climate footprint (*n* = 9341).

## 3. Results

### 3.1. Climate Impact and Energy Intake

**Figure 1:**
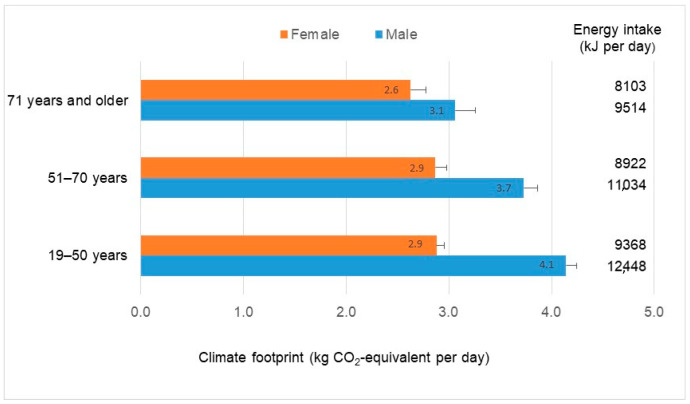
The climate footprint and energy intake of Australian adult diets based on 9341 individual daily diets reported in the Australian Health Survey. Bars show 95% confidence intervals.

The climate impacts of Australian adult diets, assessed using the GWP* climate metric, averaged 3.4 kg CO_2_-e per person per day (95% CI 3.34 to 3.44). Older females tended to have lower dietary climate impacts, averaging 2.6 kg CO_2_-e per person per day, whereas younger males tended to have higher dietary climate impacts, averaging 4.1 kg CO_2_-e per person per day (Figure 1). In part, this reflected differences in total energy intake (Figure 1). Overall, the variation between individuals in total energy intake explained around one quarter of the variation in dietary climate impacts (*R*^2^ = 0.22). This is consistent with previous studies in Australia that reported positive correlations between total dietary energy intake and dietary water scarcity footprints [30], cropland footprints [31] and dietary GHG emissions assessed using the GWP100 climate metric [29].

### 3.2. Contribution Analysis

The Australian Dietary Guidelines [26] are structured around five food groups: fruit, vegetables, grain (cereal) based foods, fresh meats and other foods rich in protein, and dairy foods (including calcium-fortified dairy alternatives). The Guidelines encourage eating a wide variety of foods from these core food groups daily. The Guidelines also acknowledge the eating of other non-core foods which are energy-dense and nutrient-poor, high in saturated fat, added sugars and salt, and alcohol. These foods, described as discretionary foods because they are not a necessary part of a healthy diet, are recommended to be eaten only sometimes and in small quantities. In addition, the Guidelines recommend the use of unsaturated spreads and oils in small quantities. Following this categorization, approximately two thirds of the climate footprint for the average Australian diets was associated with core foods, and one third associated with discretionary foods (Table 1). Healthy fats and oils and miscellaneous foods not related to any of the food groups contributed around 1%. Of the core food groups, the largest contribution came from fresh meats and alternatives (35.7%), followed by dairy foods and alternatives (11.7%), which is consistent with other studies reporting dietary GHG emissions, e.g., [5,11,12].

### 3.3. Dietary Patten Analysis

Considering the 19 to 50 year old age grouping, the current average diet was compared to a higher quality and lower climate impact diet subgroup (Table 2). This subgroup had a 45% higher diet quality score (59.4 compared to 41.0) and a 56% lower climate footprint (1.54 kg CO_2_-e per person per day compared to 3.53) than the current average diet. The most outstanding difference in dietary composition was a much lower level of intake of discretionary foods (2.44 servings compared to 7.42). There was also higher intake of most core foods. For example, fruit intake was higher at 1.90 servings compared to 1.38 servings in the current average diet. Vegetable intake was 3.71 servings compared to 2.47 servings. Intake of breads and cereals was also higher (5.73 servings compared to 4.57 servings); however, the climate impact from breads and cereals was almost half (0.15 kg CO_2_-e compared to 0.30). This was the results of lower climate impact food choices within the breads and cereals food group, especially rice, which in the Australian context has a negative climate footprint (Supplementary Table S3). Uncooked Australian-grown white rice has a climate footprint of −0.91 kg CO_2_-e/kg, compared to refined flour with a climate footprint of 0.40 kg CO_2_-e/kg, or uncooked oats with a climate footprint of 0.67 kg CO_2_-e/kg (Supplementary Table S3). Intake of fresh meats and alternatives was also marginally higher in the higher diet quality and lower climate impact subgroup (Table 2). Within this food group, there were higher intakes of seafood, poultry, pork and vegetarian alternatives, but lower intake of red meat. Overall, the climate impact associated with fresh meats and alternatives was greatly reduced (0.34 kg CO_2_-e compared to 1.23). In particular, the climate impact associated with red meat was −0.10 kg CO_2_-e compared to 0.82 kg CO_2_-e, explained by a greater preference for lamb and mutton over beef. In the Australian food system, sheep meat has a climate footprint of −4.80 kg CO_2_-e/kg (Supplementary Table S3). In comparison, meat from beef cattle has a climate footprint of 16.68 kg CO_2_-e/kg.

That said, the higher quality and lower climate impact diet subgroup still fell short of meeting the Australian Dietary Guidelines for a number of food groups. For example, the Guidelines recommend two servings of fruit per day for 19 to 50 year old adults, and five or six servings of vegetables for women and men, respectively (Table 2). If the current average 19 to 50-year diet was scaled to align with the Guidelines, with increased core food intake and reduced discretionary food intake, the climate footprint increased by around 6% from 3.53 to 3.73 kg CO_2_-e per person per day (Table 2). However, the dietary guidelines are not prescriptive about food choice within a food group, except to emphasize the importance of variety as individual foods have different micronutrient composition. If a recommended diet was based on the food choices evident in the higher-quality and lower-climate impact diet subgroup, the climate footprint decreased to 2.07 kg CO_2_-e per person per day, a reduction of around 42% compared to the current average diet of 19 to 50 year old adults (Table 2). In this lower climate footprint recommended diet, dairy foods and alternatives made the largest contribution of 0.72 kg CO_2_-e or 35% of the total. Fresh meats and alternatives contributed 0.38 kg CO_2_-e or 18% of the total, with the largest contribution coming from poultry (0.26 kg CO_2_-e). Due to the prevalence of lamb, red meat made a negative contribution to the climate footprint of the recommended diet with lower climate impact, even though there were 0.41 servings, amounting to almost three servings per week.

## 4. Discussion

In this study, a diet consistent with the Australian Dietary Guidelines was identified that had 42% lower climate footprint compared to the current average Australian adult. This demonstrates the potential for dietary change to achieve diet quality improvement as well as contribute to climate action. This improved diet was based on food choices already widely evident in the Australian community, so it should be considered realistic. That said, this diet, with a climate footprint of 2.07 kg CO_2_-e per person per day, falls short of the goal of climate neutrality.

### 4.1. Diets and the Climate Change Planetary Boundary

In recent years, planetary boundaries have emerged as an important concept in sustainability assessment [48,49,50]. This approach acknowledges that human activities inevitably have environmental impacts. However, there are absolute limits for resource use and emissions that need to be respected to avoid major and potentially irreversible earth system change. These boundaries provide important reference points for assessing the sustainability of diets. For example, it has been proposed that the global extent of croplands should not exceed 15% of the ice-free land surface [48]. Assuming these croplands are shared equitably among the current global population, a downscaled boundary for an individual daily diet is found to be around 7 m^2^ of cropland per day. Australian adult diets were previously found to marginally exceed this boundary, requiring, on average, around 7.1 m^2^ per person per day [31]. Diets with higher diet quality that required only 5.9 m^2^ of cropland per day and were therefore within the downscaled planetary boundary were also identified. Similarly, a planetary boundary for global freshwater use of 2800 km^3^ per year has been proposed [51]. On average, Australian adult diets have been found to be well within this boundary [52].

In the case of climate change, the planetary boundary was initially defined by an atmospheric CO_2_ concentration of 350 ppm or by an increase in radiative forcing, i.e., the imbalance between incoming and outgoing radiation, of 1 Wm^−2^ [48]. The latter measure is preferable as it provides scope for the inclusion of GHGs other than CO_2_, as well as other types of emissions and activities that contribute to radiative forcing [19]. In any case, these boundaries have already been transgressed. Atmospheric CO_2_ concentrations now exceed 400 ppm [53] and in 2011 the total anthropogenic radiative forcing was estimated to have reached 2.3 Wm^−2^ [18]. As such, it seems appropriate to work toward a goal whereby diets make no additional net contribution to climate change. In other words, diets should have a climate footprint, assessed using the GWP* climate metric, of less than or equal to zero.

The problem is that Australian adult diets continue to contribute to additional warming, and it is not possible to currently construct a sensible diet that is climate-neutral (i.e., GWP* ≤ 0). This is because there are presently only two types of food in the Australian food system with a negative climate footprint, i.e., products from lamb and products from rice (Supplementary Table S3). All other foods, including all Australian fruits, vegetables, dairy foods and other grains and protein foods, have a positive climate footprint.

It is possible to eat with lower climate impact, as demonstrated by the higher quality/lower climate impact diet subgroup and the recommended dietary scenario based on this subgroup (Table 2). However, there is only so much that can currently be achieved with dietary change. The real heavy lifting is needing to be done by the agricultural and food processing industries. Until such time that a large number of climate-neutral foods are available, it will not be possible for complete and nutritionally adequate climate-neutral diets to exist, other than by the independent purchase of carbon offsets from outside the food system. Therefore, it is critically important that agricultural and food producing industries are active in managing their radiative forcing footprint to achieve climate-neutrality.

The EAT-Lancet global reference diet represents a proposed dietary pattern designed to feed a future population of 10 billion while remaining within planetary boundaries [54]. However, with the EAT-Lancet global reference diet, the definition of the climate change planetary boundary excludes all CO_2_ emissions from fossil fuels and other sources. These emissions are assumed to have been reduced to zero ([54], p. 19). This is not reflected in current trends as global CO_2_ emissions from the burning of fossil fuels continue to rise and are regarded as the primary threat to the Paris Agreement and planetary health [55]. As such, it is difficult to see how the EAT Lancet global reference diet can be considered to be consistent with climate-neutral food consumption.

### 4.2. Choice of Climate Metric

As far as it is known, this is the first study to assess dietary GHG emissions using the GWP* climate metric. As such, the study provides important new evidence in relation to the climate impacts of population dietary habits. Previous studies have almost exclusively used the GWP100 climate metric which has limitations that need to be acknowledged [5]. There is no absolute equivalence in climate impact between different GHGs [19]. As an analogy, according to the Australian Food Composition database [56], calcium fortified rice beverage has a calcium content of 74 mg/100 mL and a protein content of 0.3 g/100 mL. In comparison, reduced fat milk has 120 mg of calcium and 3.7 g of protein per 100 mL. Consequently, it could be said that 162 mL of calcium fortified rice beverage is *equivalent* to 100 mL of reduced fat milk. However, this equivalency is relevant only to calcium. If another basis for equivalency was chosen, such as protein, the relationship between these two foods could be substantially different. In the same way, the GWP100 climate metric establishes equivalence between different GHGs based specifically on the integral of radiative forcing over a future 100-year time horizon. However, such a basis for equivalence is not directly relevant if the goal is climate stabilization, as expressed in the Paris Agreement, as the emissions reduction necessary depends on the particular basket of GHGs concerned [19]. Although there are now many studies, based on the GWP100 climate metric, reporting potential emissions reduction possibilities by adopting one dietary pattern over another, it is unclear what level of reduction is necessary. Even an 80% reduction in GHG emissions is not adequate to achieve climate stabilization if the remaining emissions are CO_2_ or another long-lived GHG. He Ridoutt lies the importance of the GWP* climate metric as it establishes equivalence on the basis of future warming. As such, this new metric [20,21] can be used to align food systems with the goal of climate stabilization. A detailed description of the GWP* climate metric and its limitations is available in the original source documents [20,21,42].

This study has assessed self-reported diets obtained from the Australian Health Survey using the food groups described in the Australian Dietary Guidelines [26]. The higher diet quality and lower climate footprint dietary pattern was identified from within the observed data by quadrant analysis. This study has not sought to evaluate dietary scenarios defined by the authors which could potentially introduce bias in the research design. Application of the GWP* climate metric leads to results which differ for some foods compared to results obtained with the GWP100 climate metric, most notably in the case of sheep meat and rice produced in Australia where the climate footprints were negative. This occurs when the contribution to radiative forcing from current industry emissions is less than the decrease in radiative forcing from the decay of historical emissions. In essence, these industries are no longer making any additional contribution to climate change, a status consistent with climate stabilization. The choice of climate metric in this study (GWP*) largely explains the differences compared to other GHG emissions studies of diets using the GWP100 climate metric. In this study, fresh meats and discretionary foods made the largest contribution to the climate footprint, which is consistent with results reported previously using the GWP100 climate metric [29]. However, the relative contributions of red meat, seafood, poultry, pork, and vegetarian alternatives differed marginally as the climate impacts of beef, lamb, pork, and to some degree dairy foods are sensitive to how the different gases are included in the chosen metric. In our view, greater attention needs to be given to the choice of climate metric used in dietary studies. Our concern is the seemingly uncritical application of the GWP100 climate metric. As noted previously [5], dietary GHG emission studies habitually employ the GWP100 climate metric, usually without justification for this modelling choice and consideration of the implications on study conclusions. For reasons discussed earlier in this article, the GWP* climate metric may be more relevant when addressing the challenge of climate stabilization.

### 4.3. Limitations

This study has examined the climate impact of Australian adult diets. Other environmental aspects were not addressed, and the findings cannot be used to support conclusions about overall sustainability. That said, the same 9341 daily diets have previously been assessed for water scarcity footprint [30] and cropland scarcity footprint [31] and it has been found that correlations between environmental indicators are generally weak (Pearson correlation 0.2 to 0.3) after controlling for total energy intake. Consequently, actions to reduce dietary climate impacts may not necessarily improve other environmental aspects. Further research is needed to explore pathways that enable simultaneous improvement in multiple environmental aspects with the avoidance of negative trade-offs (i.e., reducing one environmental impact while increasing another). Additionally, in common with many dietary GHG-emission studies, emissions from land use and land use change were not included due to a lack of consistent and relevant data at the level of specific foods. In addition, the study did not address food losses and waste, due to limited local and food item-specific data. This is another topic for future research.

This study used Australian dietary intake data and climate impact data relevant to foods in the Australian food system. The findings may not be relevant to other regions where dietary patterns and food production systems differ. Care was taken to select the highest quality data sources. The dietary intake data are considered high quality, having been obtained from the Australian Health Survey that employed a nationally representative sampling process and a systematic collection procedure [28]. Like all 24-h dietary recall surveys there is potential for under-reporting of foods and published estimates of the under-reporting prevalence were used to uniformly correct the data. If the under-reporting was biased towards discretionary foods, it is possible that the climate impact of these foods has been under-estimated. In estimating the climate impacts of foods, a robust published method was applied [20,21] (References [57,58,59,60,61,62,63,64] are cited in the Supplementary Materials).

## 5. Conclusions

Most evidence about the climate impact of current diets and alternative dietary strategies has arisen from studies employing the GWP100 climate metric. However, the relevance of this climate metric is questionable, especially since the results obtained with this metric cannot be directly related to the goal of climate stabilization. To address this limitation, we apply a new climate metric, GWP*, that establishes equivalence between different GHGs in terms of future warming potential. We find that Australian adult diets continue to contribute to further warming, and although healthy diets with lesser climate impact are possible, it is currently not possible to define a sensible climate-neutral dietary strategy because so few foods in the Australian food system are climate-neutral, only products from rice and from sheep meat. In summary, to bring Australian food systems into line with the Paris Agreement’s climate stabilization goal, the emphasis needs to be upon strategies across the agricultural and food processing industries to manage their radiative forcing footprints. These findings, based on the GWP* climate metric, are novel and contradict some of the common arguments about sustainable diets that tend to focus on the relative contributions of animal and plant-sourced foods. The danger in avoiding animal-sourced foods is that this may lead to trading a short-term climate benefit from reducing short-lived methane emissions with a longer-term problem of increased CO_2_ and N_2_O emissions, making climate stabilization ever more difficult. We encourage wider application of the GWP* climate metric in studies that are intended to support climate-neutral dietary transitions. In addition, future studies should evaluate climate impacts alongside other environmental impacts associated with food production. This is to avoid the development of strategies that might reduce climate impacts but lead to other types of environmental impacts increasing. Finally, we hope that this article helps to make researchers and practitioners in the nutrition and dietetics fields more informed about the variety of climate metrics and the potential for the choice of climate metric to influence conclusions about dietary patterns that support climate goals.

## Figures and Tables

**Table 1:** Contribution of different foods (%) to the climate footprint of Australian adult daily diets (*n* = 9341) assessed using the Global Warming Potential Star (GWP*) climate metric. Food groups are as defined by the Australian Dietary Guidelines [26].

Food	Male	Female	Total
Fruit	3.2	4.3	3.7
Vegetables	3.8	5.4	4.5
Breads and cereals	8.0	8.5	8.2
Fresh meat and alternatives	35.8	35.5	35.7
*Seafood*	(*3.5*)	(*4.0*)	(*3.7*)
*Beef and lamb*	(*24.0*)	(*23.4*)	(*23.8*)
*Poultry*	(*5.5*)	(*5.4*)	(*5.4*)
*Pork*	(*1.5*)	(*1.3*)	(*1.4*)
*Vegetarian alternatives*	(*1.3*)	(*1.4*)	(*1.3*)
*Other livestock products*	(*<0.1*)	(*<0.1*)	(*<0.1*)
Dairy and alternatives	11.7	13.4	12.4
Beverages	1.9	2.6	2.2
Discretionary choices	34.1	29.2	32.0
*Sugar sweetened beverages*	(*1.5*)	(*1.2*)	(*1.4*)
*Biscuits, cakes, waffles*	(*1.3*)	(*1.6*)	(*1.4*)
*Pastries and pies*	(*3.2*)	(*2.9*)	(*3.1*)
*Processed meat products*	(*17.6*)	(*13.5*)	(*15.9*)
*Dairy desserts, cream, butter*	(*2.6*)	(*2.5*)	(*2.6*)
*Fried potato and extruded snacks*	(*0.7*)	(*0.7*)	(*0.7*)
*Muesli bars, confectionary, chocolate*	(*1.4*)	(*1.8*)	(*1.6*)
*Alcoholic beverages*	(*4.9*)	(*3.8*)	(*4.4*)
*Other*	(*0.8*)	(*1.1*)	(*0.9*)
Healthy fats and oils	0.3	0.3	0.3
Miscellaneous foods	1.2	0.7	1.0

**Table 2:** Food intake (servings person^−1^) and climate footprint (kg CO_2_-e person^−1^) for current and recommended Australian adult (19–50 years old) daily diets ^1^.

Food Group	Current Diet (n = 5157)		Higher Diet Quality/Lower Climate Footprint Subgroup (n = 1068)		Recommended Diet: Average Climate Footprint Intensity Foods		Recommended Diet: Lower Climate Footprint Intensity Foods	
	Servings	Climate Footprint	Servings	Climate Footprint	Servings	Climate Footprint	Servings	Climate Footprint
Fruit	1.38	0.12	1.90	0.15	2.0	0.18	2.0	0.16
Vegetables	2.47	0.15	3.71	0.18	5.5	0.34	5.5	0.27
Breads and cereals	4.57	0.30	5.73	0.16	6.0	0.40	6.0	0.17
Fresh meat and alternatives	2.32	1.23	2.54	0.34	2.8	1.48	2.8	0.38
*Seafood*	*0.22*	*0.12*	*0.28*	*0.10*	*0.27*	*0.14*	*0.31*	*0.11*
*Beef and lamb*	*0.66*	*0.82*	*0.37*	*−0.10*	*0.79*	*0.98*	*0.41*	*−0.11*
*Poultry*	*0.74*	*0.21*	*0.87*	*0.24*	*0.90*	*0.25*	*0.96*	*0.26*
*Pork*	*0.18*	*0.05*	*0.21*	*0.05*	*0.22*	*0.06*	*0.23*	*0.06*
*Vegetarian alternatives*	*0.51*	*0.04*	*0.81*	*0.06*	*0.61*	*0.05*	*0.89*	*0.06*
Dairy and alternatives	1.46	0.45	1.29	0.37	2.5	0.77	2.5	0.72
Discretionary choices	7.42	1.15	2.44	0.25	2.8	0.43	2.8	0.29
Miscellaneous foods		0.12		0.08		0.12		0.08
Total		3.53		1.54		3.73		2.07

^1^ The recommended diet with lower climate footprint intensity foods is based on the higher diet quality/lower climate footprint subgroup. The number of servings differs marginally for men and women in the recommended Australian diet.

## Data Availability

The dietary intake data are available from the Australian Bureau of Statistics (http://www.abs.gov.au/australianhealthsurvey accessed on 15 March 2017). The climate footprints of individual foods are presented in the Supplementary Table S3.

## Supplementary Materials

The following are available online at https://www.mdpi.com/article/10.3390/nu13041122/s1, Table S1: Comparison of climate impacts using GWP100 and GWP*, Table S2: Energy use data for food processing, Table S3: Climate footprints of individual foods.
